# 1930. Quality of Life among Patients with Community-onset *Clostridioides difficile* Infection (CDI) without Subsequent Hospitalization

**DOI:** 10.1093/ofid/ofad500.090

**Published:** 2023-11-27

**Authors:** Mark A Schmidt, Jennifer L Kuntz, Elizabeth Shuster, Richard Contreras, Judy L Donald, Joanna L Barreras, Fredrick J Angulo, Holly Yu, Elisa Gonzalez, Joann M Zamparo, Ana Florea, Sara Y Tartof

**Affiliations:** Center for Health Research, Kaiser Permanente Northwest, Portland, OR; Kaiser Permanente Center for Health Research, Portland, Oregon; Kaiser Permanente Center for Health Research, Portland, Oregon; Kaiser Permanente Southern California Department of Research & Evaluation, Pasadena, California; Kaiser Permanente Center for Health Research, Portland, Oregon; Kaiser Permanente of Southern California, Pasadena, California; Pfizer, Inc., Portland, Oregon; Pfizer Inc., Collegeville, Pennsylvania; Pfizer Vaccines, Collegeville, Pennsylvania; Pfizer Vaccines, Collegeville, Pennsylvania; Kaiser Permanente Southern California, Pasadena, CA; Kaiser Permanente Southern California, Pasadena, CA

## Abstract

**Background:**

*Clostridioides difficile* infections increasingly occur among persons who are diagnosed and receive care in the outpatient setting. We explored the impact of outpatient *C. difficile* infection (oCDI) on patients’ healthcare-related quality of life (HRQoL).

**Methods:**

We conducted this prospective study of adults aged ≥ 50 years within the Kaiser Permanente Southern California (KPSC) and Northwest (KPNW) healthcare delivery systems. We identified and recruited individuals with oCDI, defined as laboratory-confirmed CDI with no hospitalization on or in 7 days after diagnosis. To assess HRQoL associated with oCDI, we administered the Cdiff32, a CDI-specific HRQoL questionnaire, shortly after oCDI diagnosis. We also administered the EQ-5D-5L, a generic HRQoL questionnaire, at baseline and approximately 60 days after oCDI to collect Visual Analog Scales (VAS) and Indices pre-infection, on the worst day of illness, and 60 days after diagnosis.

**Results:**

Our study included 213 cases. Most were female 143 (67%), 77 (36%) were 60-69 years of age, and 167 (78%) reported no prior CDI. From the CDiff32, 77 (36%) reported *pretty* or *very bad* quality of life over the past week and 80 (38%) thought their oCDI made their health condition(s) worse. Within the EQ-5D-5L, 118 (55%) reported having moderate, severe, or complete inability to complete daily activities during the worst day of illness, compared to 20% pre-infection. Cases reported a median pre-infection VAS of 80.0, compared with 44.5 on the worst day of illness (p< 0.0001 vs pre-infection) and 80.0 at 60-days of follow up (p< 0.0001 vs worst day; 0.8129 vs pre-infection). Cases also reported median Index values of 0.9 pre-infection, 0.6 on worst day of illness (p< 0.0001 vs pre-infection), and 0.9 at 60 days of follow-up (p< 0.0001 vs worst day and p=0.0081 vs pre-infection).

**Conclusion:**

Even when managed within the outpatient setting, patients experienced a significant reduction in HRQoL during their CDI episode.

**Disclosures:**

**Mark A. Schmidt, PhD, MPH**, Intercept Pharmaceuticals: Grant/Research Support|Pfizer: Grant/Research Support|Vir Biotechnology: Grant/Research Support **Jennifer L. Kuntz, MS, PhD**, Pfizer: Grant/Research Support **Fredrick J. Angulo, DVM, PhD**, Pfizer Vaccines: Employee|Pfizer Vaccines: Employee|Pfizer Vaccines: Stocks/Bonds|Pfizer Vaccines: Stocks/Bonds **Holly Yu, MSPH**, Pfizer, Inc,: employee of Pfizer, Inc|Pfizer, Inc,: Stocks/Bonds **Elisa Gonzalez, MPH**, Pfizer: Stocks/Bonds **Joann M. Zamparo, MPH**, Pfizer Inc.: Full Time Employee|Pfizer Inc.: Stocks/Bonds **Ana Florea, PhD MPH**, Gilead: Grant/Research Support|GlaxoSmithKline: Grant/Research Support|Moderna: Grant/Research Support|Pfizer: Grant/Research Support **Sara Y. Tartof, PhD MPH**, Genentech: Grant/Research Support|GSK: Grant/Research Support|Pfizer: Grant/Research Support|SPERO: Grant/Research Support

